# A rare adverse effects of COVID-19 vaccine in a patient with a latent tumor: A case report and literature review

**DOI:** 10.3389/fonc.2023.1269735

**Published:** 2023-12-05

**Authors:** Wenjing Xu, Weiqi Nian

**Affiliations:** Department of Oncology, Chongqing Hospital of Traditional Chinese Medicine, Chongqing, China

**Keywords:** cancer, G6PD deficiency, COVID-19 vaccine, adverse reaction, multisystem inflammatory syndrome

## Abstract

The 2019 novel coronavirus infection has done significant damage to the world. The effectiveness and safety of the vaccine, the most critical measure to control the epidemic, has attracted attention. In this case, we report the diagnosis and treatment of a rare patient with adverse effects of the COVID-19 vaccine who had G6PD deficiency by genetic tests. We discuss the possible impact of G6PD deficiency on COVID-19 infection and potential vaccine adverse effects. Patients with severe G6PD deficiency should be monitored for vaccine safety. This article may complement a rare mechanism of vaccine side effects and chemotherapy-related side effects.

## Introduction

The 2019 novel coronavirus infection has inflicted significant damage worldwide. The effectiveness and safety of vaccines have been the utmost concern in controlling this epidemic. Adverse reactions to the COVID-19 vaccines have been mild, and severe adverse reactions are rare. This case reports a cancer patient’s diagnosis and treatment experience with a rare adverse reaction after receiving the COVID-19 vaccine. It discusses the particular genetic influence on the response to vaccination.

## Case report

A 72-year-old male patient was admitted to the hospital for a “left inguinal mass with asthma and edema for half a year.” The patient received his first dose of the COVID-19 vaccine in early September 2021 and the second dose in mid-October 2021 (the interval was about one month and one week). About two weeks after the second dose of COVID-19 vaccine was injected (both were VECO cell vaccine, Chengdu Institute of Biological Products Co., Ltd., and the patient could not produce batch number information), the patient gradually developed a left inguinal mass, accompanied by dyspnea, palpitation, and shortness of breath after activities, edema of both lower limbs, eyelid redness, and swelling, and lacrimal gland enlargement.

**Figure 1:**
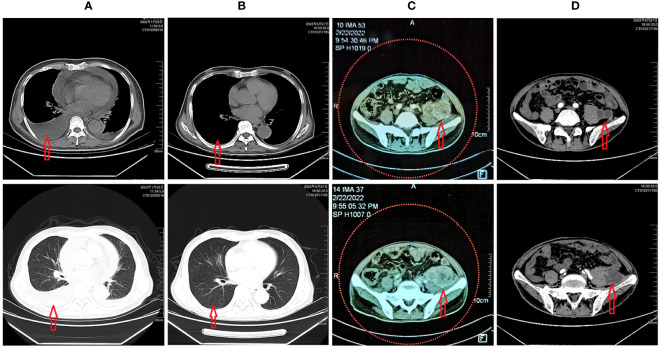
**(A)** CT scan revealed multiple serous effusions and scattered inflammation in the lungs; **(B)** After chemotherapy and targeted therapy, the patient’s multiple serous cavity effusions almost disappeared, and the lung inflammation improved; **(C)** Tumor status before chemotherapy and targeted therapy; **(D)** Tumor status after chemotherapy and targeted therapy.

The patient was repeatedly treated in the local community clinic, but symptoms were aggravated. On January 21, 2022, chest CT revealed multiple serous cavity effusions and lung inflammation (**Figure 1A**). Autoimmune-related diseases were ruled out after admission. After pleural puncture and diuresis, the patient’s symptoms were relieved. Because T-SPOT was suspiciously positive, the patient visited the public health hospital in Chongqing on February 3 for further examination to rule out tuberculosis infection. However, the patient’s symptoms of dyspnea reoccurred, and CT revealed a suspicious retroperitoneal neoplastic lesion. Therefore, the patient went to Chongqing Cancer Hospital on February 16 and underwent an inguinal lymph node biopsy.

**Figure 2:**
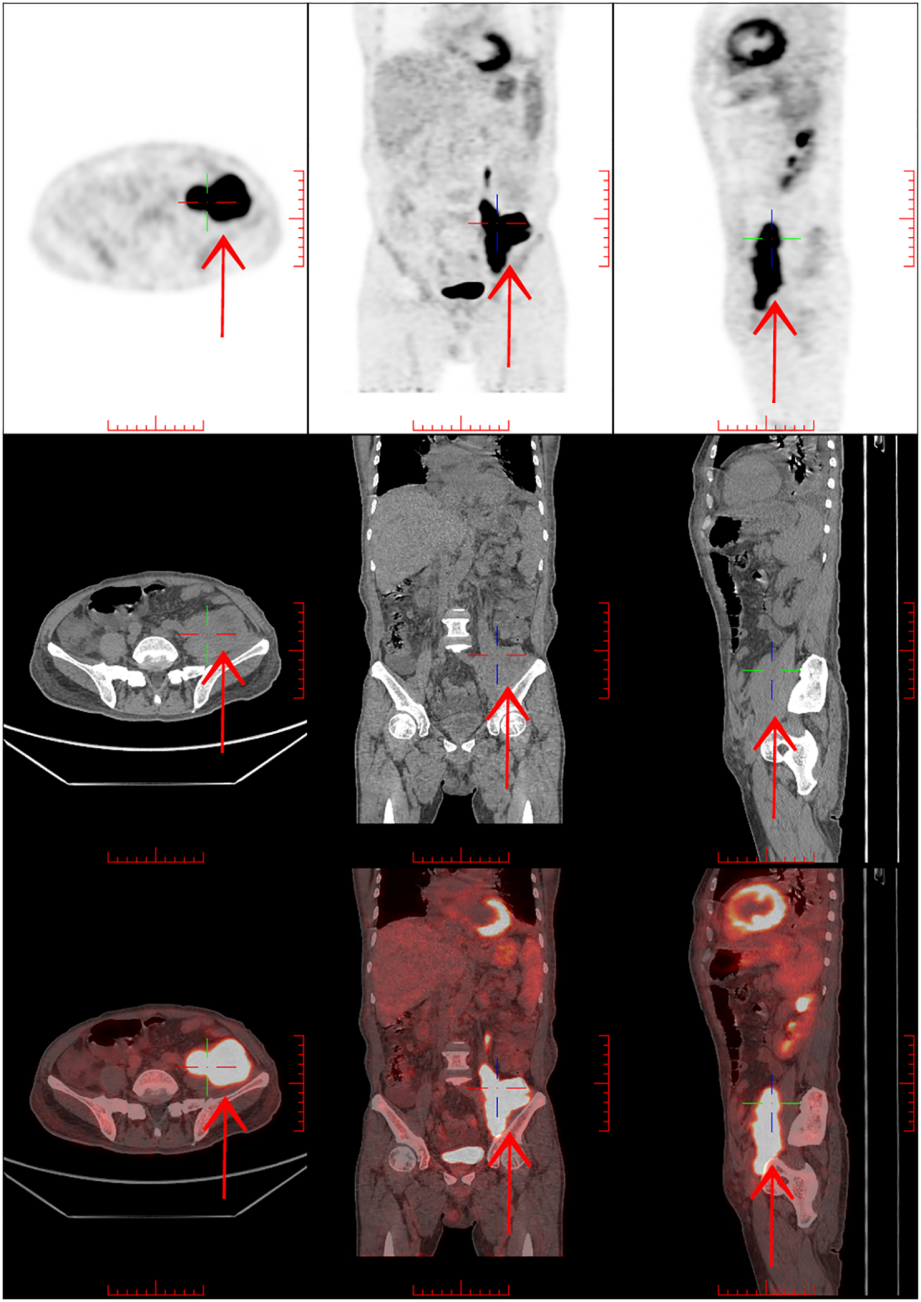
PET/CT:1. Soft tissue mass shadow in the left iliopsoas muscle area, with increased metabolism, considering mesenchymal tumor combined with pathology. 2. Lymph node metastasis in the left inguinal region is not excluded, and retroperitoneal, bilateral iliac vascular areas and right inguinal inflammatory lymph nodes may be involved. 3. Thickening and edema of fascia under the chest, abdomen, and pelvic wall. The left chest wall was thickened with increased metabolism. 4. Double lung scattered inflammation with partial atelectasis in the lower lobes of both lungs, small nodules in the upper lobes of both lungs, bilateral pleural effusion, pericardial effusion.

Pathological findings showed spindle cell hyperplasia, chronic inflammation, and focal cells with atypia. Neoplastic lesions were considered with immunohistochemistry: CK-pan (-), S-100 (-), SMA (-), STAT6 (-), CD34 (vascular +), CD68 (focal +), β-cateninmembrane (+), MUC4 (-), INI-1 (+), Desmin (focal +), RB1 (+), ALK (-), P63 (-), CD117 (individual +), MyoD1 (-), Myogenin (-), and Ki-67 (+30%). Complete lymph node excision and further biopsy were suggested. PET-CT imaging was performed on February 26, 2022 (**Figure 2**), which showed a metabolic increase in soft tissue mass in the left iliopsoas muscle area and was considered an interstitial tumor. The patient refused anti-tumor chemotherapy due to persistent wheezing, fatigue, and chest tightness and was treated with supportive therapy.

The patient came to our hospital for TCM treatment on March 14, 2022. His father, at 93, also developed multiple serous effusions after vaccination and died of secondary heart failure. Physical examination at admission: T 36.7°C, P 119/min, R 24/min, BP 94/58 mmHg, PS=3, and NRS=0. The mass was palpable, with deep pressure near the left iliac fossa with a rigid, fixed texture. The superficial lymph nodes in the left groin were enlarged approximately 2 cm, hard in texture, and immobile.

After persuading the patient to perform a needle biopsy on the retroperitoneal mass, a retroperitoneal mass spindle cell tumor was found. Immunohistochemistry showed dedifferentiated liposarcoma, and the proportion of MDM2 gene amplification was 63% (the threshold was 15%). After a series of examinations and MDT discussion in our hospital on March 24, the following diagnoses were considered: a) retroperitoneal dedifferentiated liposarcoma (T3N0-1M0 G3), b) left inguinal lymph node metastasis? c) multisystem inflammatory syndrome, d) polyserosal effusion, e) hypokalemia, f) hypoproteinemia, and g) low T3 syndrome. He was treated with daily methylprednisolone (40 mg), diuresis, albumin supplementation, and other therapies. He also received traditional Chinese medicine therapy. The patient’s dyspnea, serous cavity effusion, and both lower limbs’ edema gradually improved. Methylprednisolone was switched to daily prednisone (50 mg) and then tapered of 5 mg weekly. The patient refused to undergo further inguinal lymph node biopsy, radiotherapy, and surgical treatment and only agreed to try single-agent chemotherapy. Considering that the patient had a rare vaccination reaction, the patient was persuaded to accept gratis whole gene exome sequencing by NGS before chemotherapy (by Beijing Mygenostics Gene Technology Co. LTD). The patient received intravenous chemotherapy with epirubicin 30 mg D1-3 (PS=2 and a body surface area of 1.49 m^2^) on March 27, 2022. Moderate anemia (HGB 72 g/L) occurred on the fourth day after chemotherapy and was treated with EPO. The patient was discharged from the hospital in stable condition on April 8, 2022.

**Figure 3:**
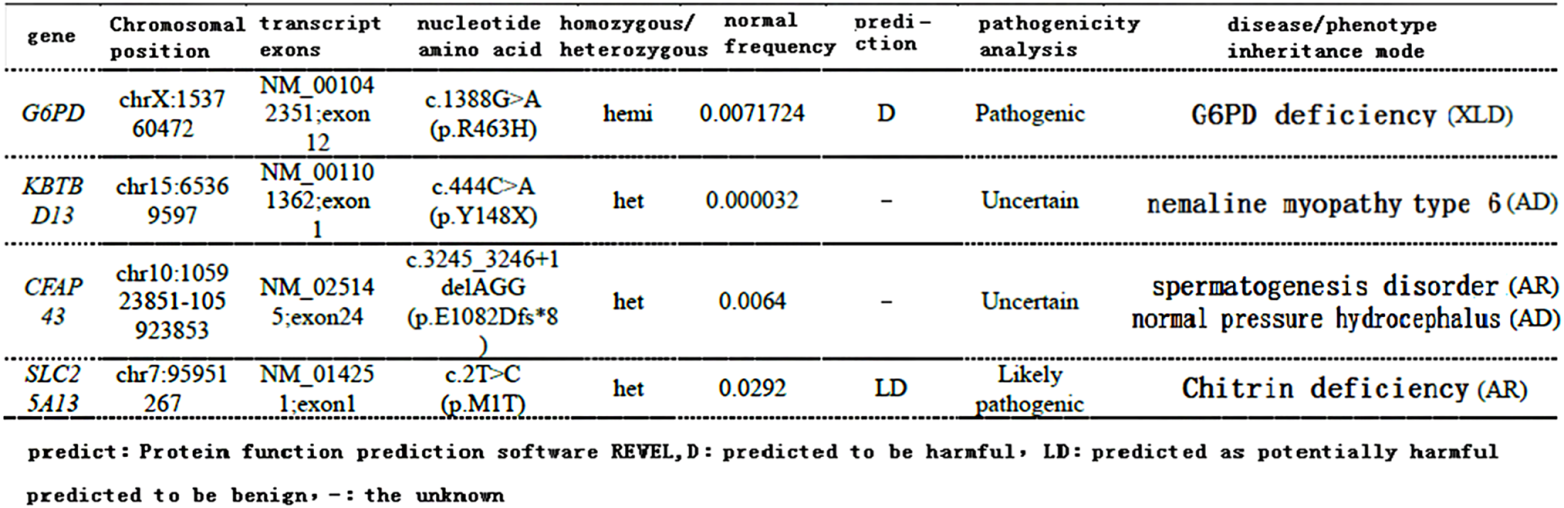
Exon part of the patient’s gene results: it is suggested that the patient has a mutation in G6PD (predicted to be deleterious).

Three days after discharge, the patient again developed edema and dyspnea, which were progressively aggravated, accompanied by fatigue and apparent anorexia. On April 13, 2022, the relevant tests suggested severe anemia (HGB 57 g/L) with serum iron of 28.5 μmol/L and reticulocyte of 0.05%. Considering the side effects after chemotherapy, the patient received a blood transfusion, an EPO injection, an iron supplement, parenteral nutrition, and other supportive treatments. The patient’s symptoms of fatigue and dyspnea were gradually relieved, and HGB increased to 86 g/L. At the same time, blood whole exome results (April 18, 2022) suggested G6DP mutation (**Figure 3**). After MDT discussion, considering the poor tolerance of chemotherapy, the patient was changed to anlotinib 12 mg d1-14 q3w. Since then, the patient’s symptoms have been stable, and no asthma, tiredness, or breast tightness occurred, and PS=1.

On May 27, 2022, the patient was re-examined during the second cycle of anlotinib. The re-examination CT showed that the tumor after treatment (**Figure 1D**) was slightly smaller than before chemotherapy (**Figure 1C**). The volume changed from 13.1 cm×6.9 cm×5.0 cm to 10.1 cm×5.3 cm×4.2 cm, which was evaluated as SD. The internal necrosis of the tumor was evident, and the enhancement was significantly weakened. At the same time, the patient’s hemoglobin returned to normal levels, and the multiple serous cavity effusion essentially disappeared (**Figure 1B**).

## Discussion

The patient was an older man born in Jiangbei district, Chongqing, with no history of anemia, jaundice, or other vaccinations. The patient recalled that he had taken fava beans and related products without discomfort and had no history of taking fava beans or particular drugs before and after the onset of this disease. The patient had an undiagnosed occult tumor when receiving the COVID-19 vaccine. Still, after the second vaccination, he developed conjunctivitis, increased heart rate, tachypnea, lung inflammation, multiple serous cavity effusion, multiple lymphadenopathies, elevated neutrophils, elevated C-reactive protein, and other manifestations of multisystem inflammatory syndrome.

This adverse effect of vaccination is relatively rare in practice, but it is similar to the multisystem inflammatory syndrome caused by COVID-19 infection (1). In recent years, there have also been reports of symptoms of a systemic inflammatory response after COVID-19 vaccination (2) and lymphadenopathy (3). These adverse reactions were observed mainly in the adenovirus vaccines. The safety of inactivated vaccines in China is relatively good, and common side effects are pain, fatigue, headache, and fever at the injection site. The symptoms are mild and generally self-limiting. A large cross-sectional survey in the UAE has also confirmed this statement (4). The China Center for Disease Control and Prevention reported that from December 15, 2020, to April 30, 2021, China administered 265 million vaccinations, and 188 abnormal serious adverse reactions were reported, with an incidence of only 0.07/100000 (5). Older people are not contraindicated to receive COVID-19 vaccination. In contrast, due to the high risk of neocoronal infection and serious complications, they need more protection from vaccines (6). However, vaccination must be avoided in patients with uncontrolled epilepsy, other serious neurological diseases, and acute and chronic active diseases. Attention should be given to selecting vaccine types and patients who lack immune protection or low immunity, such as older adults.

The patient had this rare reaction after vaccination and severe bone marrow suppression after the first low-dose single-agent chemotherapy. Considering his father’s medical history, the patient may have a unique genetic background, and the G6PD mutation was found in the follow-up whole exome testing. About 400 million people in the world are affected by G6PD deficiency. The carrier rate of G6PD gene deficiency is about 4-15%. G6PD deficiency is high in south China and south of the Yangtze River Basin. In Hubei Province, the incidence of G6PD deficiency is only 1.24%, of which about 50% is caused by the WHO type III mild disease. The clinical symptoms are not apparent (7, 8). Recent large-scale studies have shown that G6PD deficiency may lead to increased rates of COVID-19 infection and hospitalization due to severe infections and an increased risk of long-term COVID-19 infection (9). Previous studies have found that G6PD deficiency blocks the metabolism of pentose phosphate in cells. The production of NADPH and GSH is insufficient, weakening of the body’s antioxidant capacity and the host’s susceptibility to bacteria and viruses. Several studies have shown that G6PD-deficient cells are more susceptible to COVID-19 infection than normal cells (10). G6PD deficiency is associated with increased neutrophil extracellular traps (NETs), reactive oxygen species (ROS), and decreased IL-1β and NLRP3, which can lead to increased viral load. The G6PD deficiency patients may suffer heightened infectivity.

Additionally, worsened prognoses and more severe complications of infection may present in type I G6PD-deficient individuals (11). Viral infection can trigger the body’s pro-oxidant (PO) response, producing ROS to attack pathogens, a normal innate defense mechanism. However, an antioxidant system (AO) can neutralize and regulate this defense mechanism (12). COVID-19 infection can interfere with the AO system by inhibiting the production of nitric oxide (NO) in the renin-angiotensin system (RAAS). Virus-induced reduction of NO and increased aldosterone (ALD) (imbalance of NO/RAAS) lead to excessive COVID-19 immune response, manifested by fever and blood system complications, hemolysis, and thrombosis risk. Elevated ALD may lead to pulmonary edema and sodium retention. In patients with G6PD mutation, the mechanism of NO and glutathione is impaired, which causes the impairment of the AO system.

In contrast, the abnormal response of the PO system leads to the abnormal severe cytokine storm response (13). G6PD is essential for an adequate immune response, and G6PD deficiency destroys the AO system through the same pathway as COVID-19 (14). Most antibody production in conjugate vaccine injection occurs after the second vaccination (15). Therefore, it is presumed that after the patient received the second vaccination, the G6PD deficiency may lead to cytokine storms induced by an immune response. This causes the patient to develop severe systemic inflammatory response syndrome, different from type I allergy (allergic reaction) produced by the first vaccination. At present, there are case reports of acute hemolysis after vaccination in patients with G6PD deficiency and thalassemia, which is considered to be due to oxidative stress reaction after vaccination (16).

Although it is only a case report, we should monitor the vaccine safety for patients with G6PD deficiency or in areas with a high incidence of G6PD deficiency. According to the degree of enzyme deficiency and the severity of the hemolytic response to oxidative stress, the WHO classified G6PD deficiency into five different variants. Classes I to III have enzyme deficiencies associated with a hemolytic response, considered clinically relevant. Although no reports have explicitly revealed the relationship between G6PD gene mutations and COVID-19 infection and vaccine adverse effects, the different mutations in the G6PD gene may lead to different severity of G6PD deficiency and play a role in the severity spectrum of COVID-19 disease or vaccination-related adverse reactions. We suggest that G6PD screening and genetic counseling should be carried out in areas with a high prevalence of G6PD deficiency and that drug and vaccine safety monitoring should be emphasized in patients with unexplained rhabdomyolysis, hemolytic anemia, severe favism, males with familial G6PD deficiency, and class I G6PD deficiency. Free genetic tests can be established for G6PD deficiency patients with severe adverse drug or vaccine reactions, and the relevant data should be uploaded to the appropriate gene bio-bank. Establishing a gene bio-bank in the future will help researchers further analyze the relationship between specific G6PD mutations and COVID-19 disease and vaccine adverse reactions and ensure the medication safety of the G6PD deficiency patients.

Second, this patient developed severe anemia at a lower chemotherapy dose, possibly related to G6PD deficiency. We conducted a literature search but did not find the exact relationship between G6PD deficiency and the safety of chemotherapy drugs. Previous studies have found that G6PD-deficient RBCs are challenging to deal with oxidative stress and are more prone to destruction, producing dominant or recessive hemolysis (17). The mouse animal models show that G6PD deficiency enhances benzene-induced dysfunction of bone marrow hematopoietic stem cells and hematopoietic progenitor cells (18). This suggests that G6PD deficiency may lead to the aggravation of chemotherapy side effects. However, some studies have reported that germ-cell tumor patients (19) and breast cancer patients (20) with G6PD deficiency do not develop significant adverse myelotoxicity during chemotherapy. 

There are more than 140 variant types in G6PD, and the WHO classifies G6PD defects into I-V categories (21). The clinical symptoms of different types of patients are significantly different. The G6PD gene is not routinely tested in chemotherapy patients. Hence, the connection between G6PD deficiency and chemotherapy adverse reactions deserves more research. G6PD is a vital enzyme of the pentose phosphate pathway (PPP). Its main product, NADPH, is essential for regenerating glutathione (GSH), crucial in cellular antioxidant defense. At the same time, NADPH also plays various roles in redox signal-mediated cell regulation, such as NADPH oxidase (NOX) and nitric oxide synthase (NOS) produce ROS and reactive nitrogen species (RNS), respectively. Maintenance is an essential messenger for redox homeostasis regulation (10). G6PD deficiency may have many adverse effects on the human body, such as anemia, hemolysis, inflammatory infection, cardiovascular disease, insulin resistance, and immune deficiency (22). However, it has positive aspects for anti-tumor treatment. The abnormal increase in G6PD activity can improve the proliferation of tumor cells, stimulate tumor angiogenesis, and increase the resistance to radiotherapy and cytotoxic drugs (23).

In contrast, G6PD deficiency inhibits tumor growth and proliferation and increases apoptosis and the response of tumors to cytotoxic drugs (24). However, anlotinib can up-regulate the expression of NADPH oxidase 5 (NOX5), increase the production of ROS, impair mitochondrial respiration, and promote tumor cell apoptosis (25). Whether the anti-tumor effect of anlotinib can be increased in tumor patients with G6PD germline mutations and have decreased oxidative stress ability deserves further investigation.

Finally, as this case shows, diagnosing and typing sarcoma in soft tissue is challenging, and sufficient histological evidence is needed. Definite tissue acquisition and pathological characteristics are the premises of definitive diagnosis. Therefore, lymph node complete excisional biopsy has more diagnostic priority than needle or aspiration biopsy, and the primary site is more representative than the biopsy tissue of the metastatic site.

## Conclusions

We report a case of rare adverse effects of the COVID-19 vaccine with a latent tumor. Moreover, the results of the targeted NGS, G6PD gene deficiency, were less reported. Therefore, our case enriches the clinical and genetic scales of the COVID-19 vaccine’s adverse effects.

## Data availability statement

The datasets presented in this study can be found in online repositories. The names of the repository/repositories and accession number(s) can be found in the article/supplementary material.

## Ethics statement

The requirement of ethical approval was waived by Ethics Committee of Chongqing Hospital of Traditional Chinese Medicine for the studies on humans because blood sampling, genetic testing and treatment of patients basically meet the requirements of treatment routine and guidelines. The patient has signed the relevant informed consent. The studies were conducted in accordance with the local legislation and institutional requirements. Written informed consent for participation was not required from the participants or the participants’ legal guardians/next of kin in accordance with the national legislation and institutional requirements. The human samples used in this study were acquired from a by-product of routine care or industry. Written informed consent was obtained from the individual(s) for the publication of any potentially identifiable images or data included in this article.

## Author contributions

WX: Writing – original draft. WN: Writing – review & editing.
